# The gutSMASH web server: automated identification of primary metabolic gene clusters from the gut microbiota

**DOI:** 10.1093/nar/gkab353

**Published:** 2021-05-21

**Authors:** Victòria Pascal Andreu, Jorge Roel-Touris, Dylan Dodd, Michael A Fischbach, Marnix H Medema

**Affiliations:** Bioinformatics Group, Wageningen University, 6708PB, Wageningen, The Netherlands; Bijvoet Centre for Biomolecular Research, Faculty of Science – Chemistry, Utrecht University, 3584CH, Utrecht, The Netherlands; Department of Pathology, Stanford University, Stanford, CA 94305, USA; Department of Microbiology & Immunology, Stanford University, Stanford, CA 94305, USA; Department of Microbiology & Immunology, Stanford University, Stanford, CA 94305, USA; Department of Bioengineering, Stanford University, Stanford, CA 94305, USA; Chan Zuckerberg Biohub, San Francisco, CA 94158, USA; Bioinformatics Group, Wageningen University, 6708PB, Wageningen, The Netherlands

## Abstract

Anaerobic bacteria from the human microbiome produce a wide array of molecules at high concentrations that can directly or indirectly affect the host. The production of these molecules, mostly derived from their primary metabolism, is frequently encoded in metabolic gene clusters (MGCs). However, despite the importance of microbiome-derived primary metabolites, no tool existed to predict the gene clusters responsible for their production. For this reason, we recently introduced gutSMASH. gutSMASH can predict 41 different known pathways, including MGCs involved in bioenergetics, but also putative ones that are candidates for novel pathway discovery. To make the tool more user-friendly and accessible, we here present the gutSMASH web server, hosted at https://gutsmash.bioinformatics.nl/. The user can either input the GenBank assembly accession or upload a genome file in FASTA or GenBank format. Optionally, the user can enable additional analyses to obtain further insights into the predicted MGCs. An interactive HTML output (viewable online or downloadable for offline use) provides a user-friendly way to browse functional gene annotations and sequence comparisons with reference gene clusters as well as gene clusters predicted in other genomes. Thus, this web server provides the community with a streamlined and user-friendly interface to analyze the metabolic potential of gut microbiomes.

## Graphical Abstract

**Graphical Abstract:**
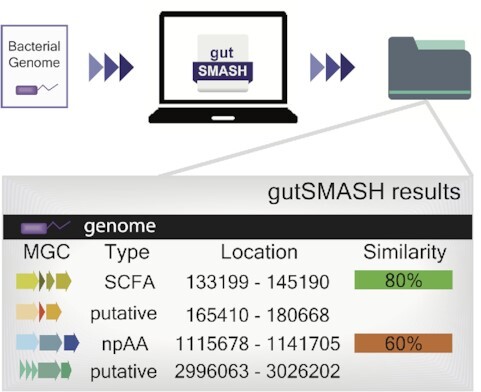
Overview of the gutSMASH pipeline for identification of primary metabolic gene clusters.

## INTRODUCTION

Microbiome research has received considerable attention in the last decade, charting the taxonomic and functional diversity found in complex ecosystems and the effects on their host. Many microbiome-associated phenotypes in the gut microbiome are derived from small molecules synthesized by anaerobic bacteria (1). These compounds are mostly products of their primary metabolic pathways that allow them to either colonize specific micro-niches in the gut or interact with other microbes. Despite the fact that some of these molecules are produced by low-abundance bacteria, they can reach high concentrations in the gut as well as in blood plasma, sometimes comparable to those of therapeutic drugs (2). This is of great interest because these molecules can profoundly modulate host metabolism, immunity, and homeostasis. An example of such an end product is trimethylamine, derived from carnitine and choline metabolism, which is known to be associated with increased risk of cardiovascular disease (3). Another interesting pathway is ethanolamine utilization, which has been indirectly associated with bacterial pathogenesis by enabling these bacteria to bloom, using this molecule as carbon and nitrogen source (4). Finally, short chain fatty acids (SCFAs) have been found to positively impact human health, their most abundant representatives are acetate, butyrate and propionate. Among other functions, butyrate is used by colonocytes as their main energy source and promotes cell differentiation (5).

Characterizing the metabolic potential of the human microbiome will enable the research community to define how metabolic interactions among microbes and with the host influences human health. Although tools exist to predict the metabolic potential of bacteria, they are either focused on predicting gene clusters encoding the biosynthesis of secondary metabolites (e.g. antiSMASH (6)) or they identify only individual genes instead of MGCs. Moreover, these tools strongly depend on generic primary metabolic databases such as KEGG (7) or BioCyc (8) that do not always include such specialized primary metabolic pathways and only cover known pathways (9). Examples of such tools for studying primary metabolism are the HUMAnN pipeline (10), MetaPath (11) or MetaTrans (12). Given the fact that genes encoding for specialized primary metabolic pathways are often found clustered together and evidence exist that the metabolic potential of such bacteria is far from fully uncovered (13), we recently introduced gutSMASH (9), a tool that identifies known as well as potentially novel MGCs, based on the antiSMASH version 5.0 framework (14).

Here, we present the gutSMASH web server, available at https://gutsmash.bioinformatics.nl/, which is designed to mine anaerobic bacterial genomes for primary specialized metabolic gene cluster (MGCs) in a user-friendly manner. The server is able to predict not only known MGCs but also putative gene clusters that may aid the discovery of novel molecules of importance for human (or animal) health. Moreover, gutSMASH also predicts gene clusters related to energy acquisition. This platform runs the algorithm on any given correctly formatted genome the user inputs, and outputs an interactive visualization that provides information on the predicted gene cluster. Additionally, it performs comparative genomic analysis using two customized databases (KnownClusterBlast and ClusterBlast) to assess the novelty of the predicted MGCs, assess their taxonomic distribution and identify architectural variants. Also, it annotates MGC genes into functional categories and highlights these with specific colors. In order to provide examples on the use of gutSMASH, we present an analysis of several genomes to showcase the diversity of gene clusters that this tool can predict, their annotations and their similarity to any gene cluster previously predicted by gutSMASH.

## MATERIALS AND METHODS

### Overview of the gutSMASH workflow

**Figure 1.**
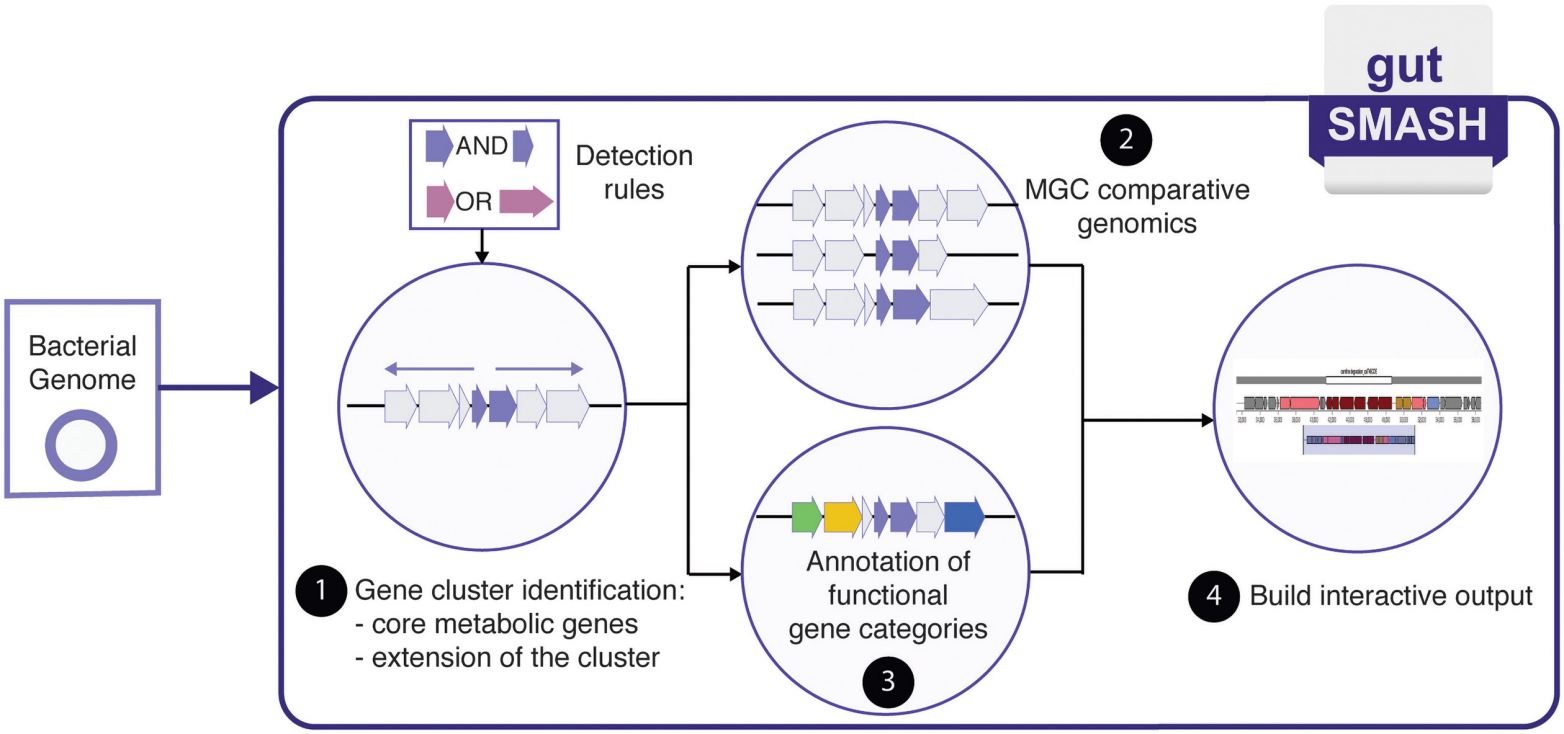
Overall workflow of the gutSMASH web server. gutSMASH takes a bacterial genome sequence as input either in GenBank, EMBL or FASTA format. First, the program iterates over the detection rules to identify the gene clusters. Next, if enabled, the predicted MGCs are compared to the databases specified to evaluate the similarity to any known pathways, or to assess the similarity to gene clusters that were predicted by gutSMASH from publicly available whole-genome sequences.

The gutSMASH algorithm is based on the antiSMASH version 5 framework. As in antiSMASH, detection rules are used for MGC identification, consisting of Pfam combinations that constitute the signature of a given metabolic pathway. The design and validation of the detection rules are described in detail in Pascal Andreu *et al.* (9). Figure 1 illustrates the different steps followed by gutSMASH. From a bacterial genome specified by the user, gutSMASH first identifies the core metabolic genes by iterating over the detection rules. Once the core genes are identified, each protocluster is extended from each flank to include accessory genes. Then, if the ‘KnownClusterBlast’ and/or ‘ClusterBlast’ options are enabled, gutSMASH performs an MGC comparative genomic analysis by blasting the predicted gene clusters to a collection of known and characterized MGCs and/or to a broader collection of gutSMASH-predicted MGCs respectively. Next, if desired, gutSMASH can functionally annotate genes into eight different categories: core biosynthetic, additional biosynthetic, transport-related, regulatory, resistance, and others (already found in antiSMASH) and encapsulation- and electron-transport-related genes as new additions. Once all the analyses have finished, gutSMASH writes the results and displays the interactive output. Also, the web server gives the option to download all results as a ZIP file.

### Interactive input and output

The ideal input for gutSMASH is an annotated nucleotide file in Genbank or EMBL format. The user can either upload a GenBank/EMBL file manually, or simply enter the GenBank assembly accession number, upon which gutSMASH will automatically use the annotated assembled genome from the NCBI FTP. Alternatively, the user can provide a FASTA file containing one or more sequences. In this case, gutSMASH will predict the genes and annotate the genome using Prodigal (15), and use those annotations to run the rest of the analysis.

The gutSMASH results can either be visualized online in a browser or downloaded locally. The output consists of several interactive HTML pages that allow the user to explore the results further. The overview page gives information on all the predicted MGCs, including their location in the genome and the functional class to which each of the MGCs belong. The main page also contain links to the gutSMASH documentation page (https://gutsmash-documentation.readthedocs.io/en/latest/) for more details. Moreover, each predicted MGC can be visualized individually for further inspection of additional MGC-specific results, depending on the options enabled before submitting the job. Besides the HTML pages, gutSMASH also generates plain text files with the KnownClusterBlast/ClusterBlast results (more details in section *Comparative genomic analysis to identify distant homologs and assess MGC taxonomic distribution*) and a GenBank file for each predicted region for further processing.

### Comparative genomic analysis to identify distant homologs and assess MGC taxonomic distribution

GutSMASH uses two different databases, KnownClusterBlast and ClusterBlast, to find MGCs homologous to the query. This comparative analysis can provide good indications of the distribution of the MGC among bacterial taxa from the human microbiome, provide insights into the extant variation of MGC architectures (gene content) and provide clues regarding MGC function (using homology-based inference).

The KnownClusterBlast module aims to identify similarities between the predicted MGCs and a reduced set of genetically and biochemically characterized gene clusters. In order to design the detection rules for these known pathways, a reduced set of well-known pathways was analysed [as described in (9)]. The sequences of these MGCs of known function were included in the KnownClusterBlast database, which currently contains 59 entries. Therefore, ticking the KnownClusterBlast button allows the user to identify which MGCs are homologous to and likely share the same function with those reference MGCs, and to study the similarities and differences in detail. Given its usefulness, this option is enabled by default.

To build the ClusterBlast database, the Culturable Genome Reference (CGR) collection (16), the Human Microbiome Project (HMP,https://www.hmpdacc.org/reference_genomes/reference_genomes.php) reference genomes and 414 Clostridiales complete genomes available in October 2019 under taxid 186802 were used as input for gutSMASH. Based on the output, both the known and putative predicted-gene clusters, a total of 30,883 MGC selected sequences, were combined to form the ClusterBlast database. When the ClusterBlast option is enabled, DIAMOND (17) is used to compare the predicted MGC protein sequences to those in the ClusterBlast MGC database to identify close homologs, using the same procedure as in antiSMASH. After ranking the gene clusters based on the numbers of pairs of homologous proteins and the cumulative bit scores, the top 10 most similar gene clusters (based on highest bit scores) are displayed in the ClusterBlast HTML tab of each predicted region. The complete list of homologs that includes similarity scores can be retrieved from the ClusterBlast output folder in the downloadable ZIP output.

### Annotation of functional gene categories: additional pmDBFA categories

The module ‘primary metabolite domain-based functional annotations’ (pmDBFA) (analogous to the secondary metabolite Clusters of Orthologous Groups [smCOGs] in antiSMASH) facilitates the functional annotation of accessory genes within a predicted gene cluster into different categories based on the presence of key Pfams in the gene-coding sequences. To tailor these annotations towards a more meaningful classification for gutSMASH output, two extra functional categories were included: encapsulation and electron transport-related genes. There are several Pfams that belong to these categories, such as the Electron transfer flavoprotein FAD-binding domain (PF00766) or the BMC domain (PF00936), respectively, which are regularly found in pathways predicted by gutSMASH. For instance, in the reductive metabolism of aromatic amino acids to arylpropionates, an electron transfer protein encoded by *etfA*, which harbours an electron-transferring flavoprotein domain (PF00766), is required to reduce the substrate (18). The encapsulation category in contrast, aims to include genes involved in bacterial microcompartmentalization, which have been found to be important for some reactions, such as those catalyzed by the enzymes encoded by the propanediol utilization operon (*pdu*), to encapsulate pathway intermediates that would be toxic for the cell at high concentrations (19). Nevertheless, in some cases, these domains are part of the detection rules of known pathways, and thus are also annotated as core genes. When found in the flanking regions, they are annotated in the corresponding category.

### Code development and server implementation

The gutSMASH server, hosted at https://gutsmash.bioinformatics.nl/, is based on the Python3 Flask web framework (https://flask.palletsprojects.com) for server-side logic combined with Jinja templating language (https://jinja.palletsprojects.com) and JavaScript for client-side logic. The submission interface requires different (some of them optional) inputs from the user, including a mandatory valid sequence file or assembly accession ID. For the job handling, different statuses were defined as:

*Submitted*: The job has been successfully submitted.
*Queued*: The job is waiting to be processed.
*Running*: gutSMASH analysis have started.
*Finished*: The job has successfully finished.
*Failed*: An error has occurred, and it is displayed for troubleshooting.
*Notified*: The job has successfully finished, and the user has been notified.
*Failed-notified*: An error has occurred, and the user has been notified.

To handle all the job statuses, the Advanced Python Scheduler (APScheduler) library was used. Please note that the status *notified* and *failed-notified* are only applicable if the user provides an email address. Finally, the communication between the web interface and the application is done via a Redis database that stores the job's information. As reference, the antiSMASH (14) (https://github.com/antismash/websmash/tree/master/websmash) and plantiSMASH (20) (https://github.com/plantismash/webserver) web servers were used for inspiration for the main layout and content.

## RESULTS

In the following sections, we provide several examples to illustrate how gutSMASH works and how to interpret the results.

### gutSMASH detects known and putative gene clusters from prominent human gut pathogens

**Figure 2.**
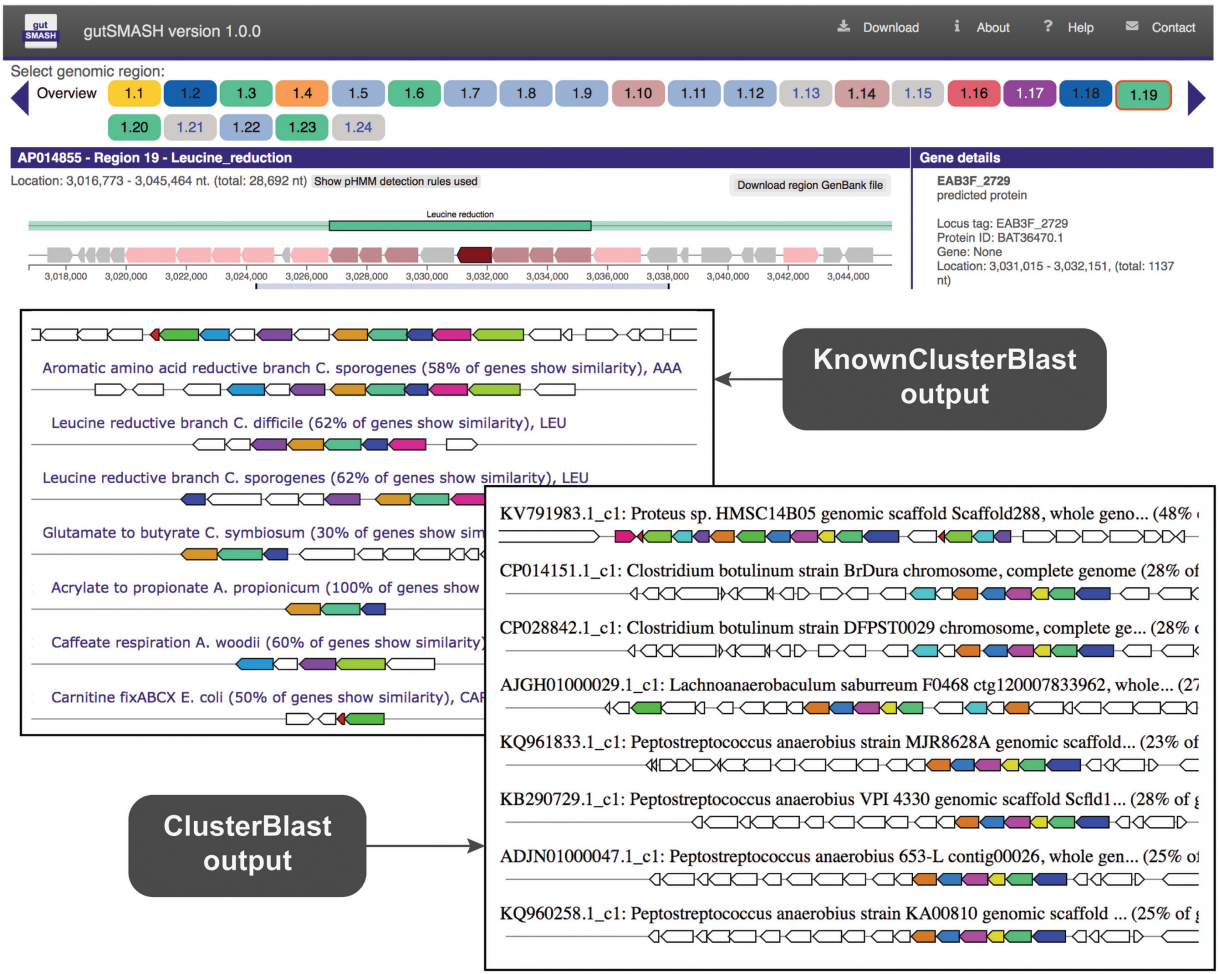
gutSMASH run for *Escherichia albertii* (GCA_002285455.1). From 24 predicted MGCs, the leucine reductive branch MGC is highlighted as an example (region 1.19). The KnownClusterBlast results show that five coding genes of this MGC share similarity with the leucine reductive branch reference gene cluster genes (overall 62% similarity) and seven with the aromatic amino acid reductive branch ones (overall 58% similarity). The ClusterBlast output shows that this gene cluster does not have homologous MGCs among other *Escherichia* members present in the database.

*Escherichia albertii* belongs to the family *Enterobacteriaceae* and is an emerging enteropathogen (21). As a close-relative of *Escherichia coli*, certain metabolic functions are expected to be shared while others may differ. To uncover its specialized primary metabolism and bioenergetics, we used the GenBank assembly accession (GCA_002285455.1) as input to the gutSMASH server and found that this genome contains 24 MGCs that belong to different MGC classes (see Figure 2). From these, 19 gene clusters have 50% or higher overall gene cluster similarity to reference MGCs when using the KnownClusterBlast database as reference. Among them, gutSMASH identifies homologs of the known carnitine (*cai*) degradation operon (22) (100% identical), the propanediol utilization (*pdu)* operon (23) (91% identical), the ethanolamine utilization (*eut)* operon (4) (94% identical) and the threonine to propionate degradation pathway (24) (90% identical). Enabling the ClusterBlast option also allowed to check whether other bacteria shared similar gene clusters or not. As expected, most of the predicted MGCs are found in other *Escherichia* genomes. However, the leucine reductive branch gene cluster (62% identical to the leucine reductive branch reference MGC) is rarely found among other close relatives, as shown in the ClusterBlast results in Figure 2.

**Figure 3.**
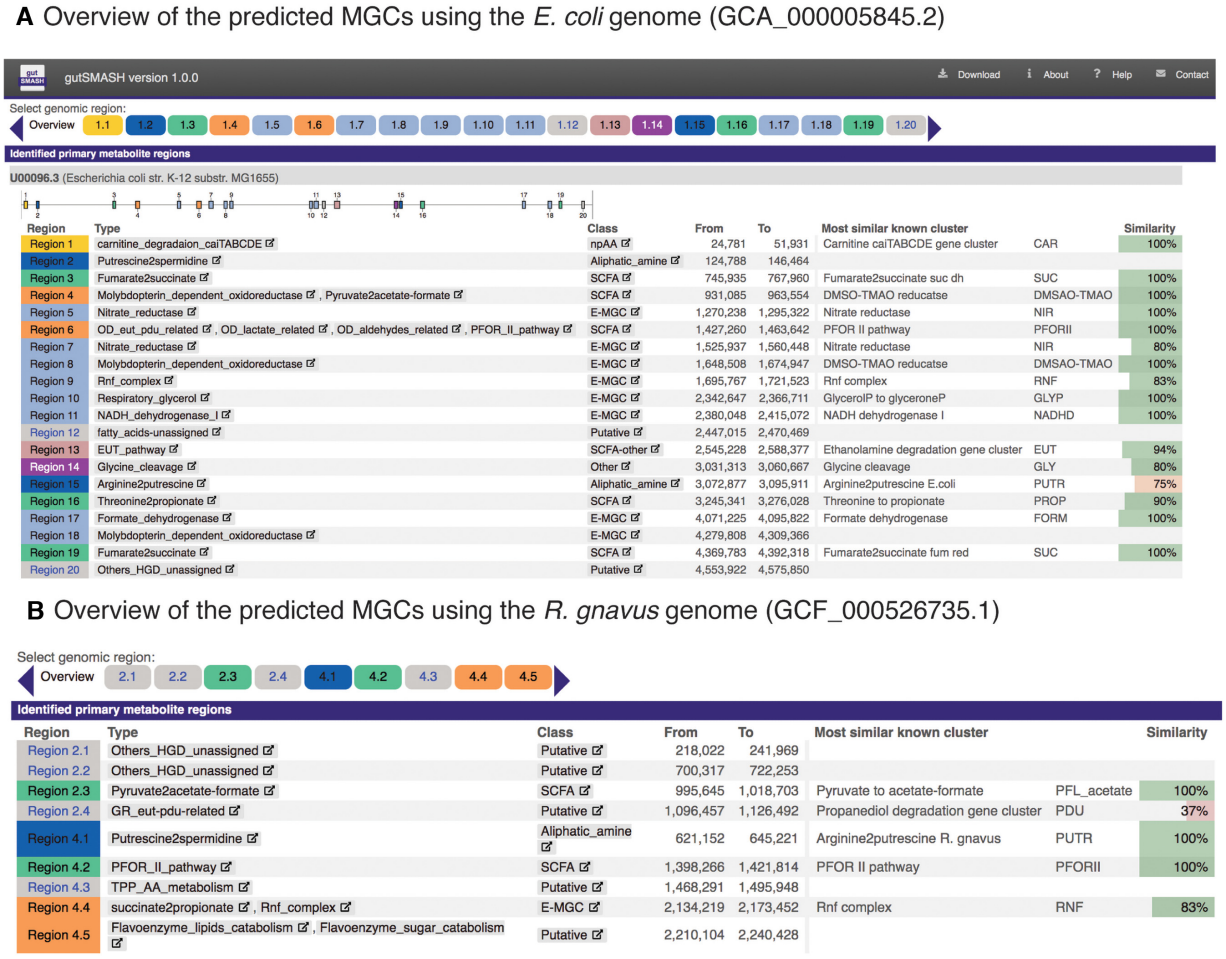
Overview of the gutSMASH runs for the (**A**) *E. coli* K-12 and (**B**) *R. gnavus* genomes. In the overview, various pieces of information regarding the predicted MGCs can be seen, such as the number of predicted MGCs, type and class, and genomic coordinates. If there is similarity to any MGC from the KnownClusterBlast database, the overall percentage similarity (percent of genes in the reference MGC with a homolog in the query) can also be seen.

For comparison, we also analysed the genome of *Escherichia coli* K-12 (GCA_000005845.2) and found that this genome contains 20 MGCs, 16 of them with overall gene cluster identity of ≥75% with known reference MGCs, when compared to the KnownClusterBlast database (see Figure 3A). Among the predicted MGCs, we find the *cai* and *eut* operon, but the lacks the *pdu* and the leucine reductive branch MGC, among others, when compared to *Escherichia albertii*.

To also show an example from a different phylum, we analysed a genome of *Ruminococcus gnavus*, a firmicute species that has been previously associated with Crohn's disease for the secretion of a complex polysaccharide that promotes inflammation (25). In this case, the genomic FASTA file of the strain AGR2154 (GCF_000526735.1) was used as input for gutSMASH, which predicted 10 MGCs (in 9 regions, with Region 4.5 containing two MGCs; see Figure 3B). Five of them were classified as putative, since they either do not have matches to any entry in the KnownClusterBlast database or the overall sequence identity is very low. Interestingly, this microbe produces the aliphatic amine spermidine that acts as an anti-inflammatory agent by suppressing immune reactions (26).

### Gene functional annotations help to get more insights on the reaction

**Figure 4.**
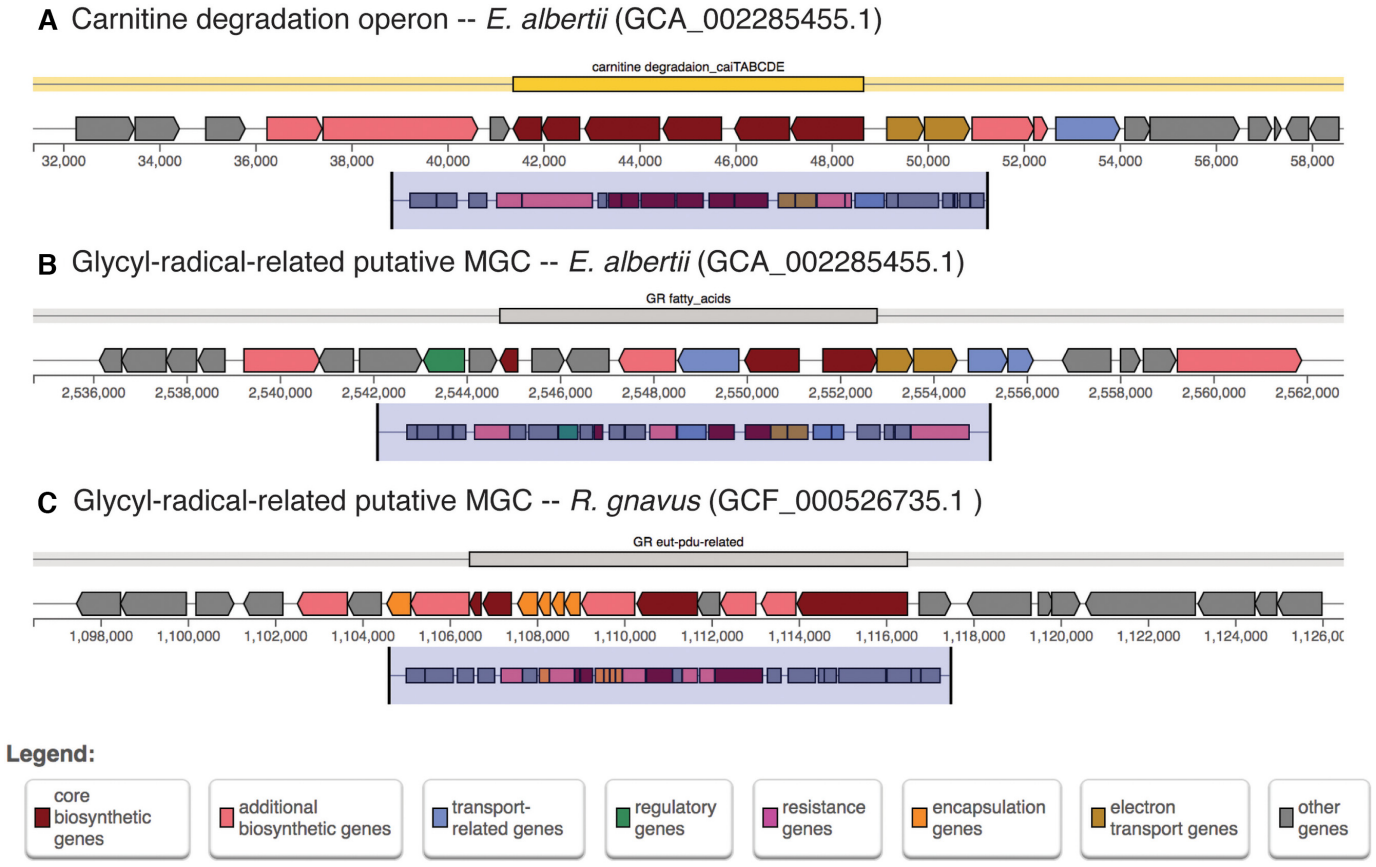
Known and putative MGCs predicted by gutSMASH with functionally annotated genes. A and B are predicted from *E. albertii* and C from *R. gnavus*. Gene cluster A represents a carnitine degradation operon, thus a known MGC, which contains core (dark red arrows) and auxiliary genes (salmon) but also transport-related (blue) and electron transport-related genes (dark yellow). Gene cluster B is a putative gene cluster that encodes a glycyl-radical enzyme. The MGC also contains genes belonging to the following categories: auxiliary, electron transport and regulatory. Gene cluster C is a glycyl-radical-related putative MGC with several encapsulation-related genes.

In the three gutSMASH runs, we enabled the gene functional annotations using the pmDBFA module. From the results, we could confirm that known MGCs such as the *cai* operon encode for two electron-transfer proteins in *E. albertii* (BAT33784.1 and BAT33785.1), which may participate in electron transfer reactions to fuel the electron transport chain. However, we also find these coding genes in other MGCs of unknown function, such as the predicted glycyl-radical (GR) fatty-acids gene cluster (see Figure 4A and B). The latter, belongs to the GR class since it encodes for a pyruvate formate-lyase but also for an acyl-CoA dehydrogenase. The gene color annotations help to visualize and show that this putative MGC not only codes for the core enzyme-coding genes but also for transport, regulatory and electron-transport related genes. Similarly, for the *Ruminococcus gnavus* genome, we find a putative MGC that belongs to the GR class since the coding sequence of *ctg2_1090* harbours a Gly_radical and PFL-like domain (PF01228 and PF02901 respectively, see Figure 4C). One of the genes also codes for an aldolase (PF00596) and, because of the presence of several BMC-coding genes, it indicates that this pathway might be encapsulated into the so-called bacterial microcompartments.

## CONCLUSION AND FUTURE PERSPECTIVES

Currently, gutSMASH is able to predict a wide range of known and putative gene clusters that are interesting to functionally profile gut microbiomes, and, in principle, any other microbiomes where the profiled pathways regularly occur, including skin and oral. The set-up of this user-friendly web server makes this tool accessible to researchers that are not familiar with the command line. Moreover, the examples provided in this article show how, by enabling the comparative genomics analysis and the functional gene annotation options, the user can extract useful information to interpret and further analyse the predicted regions.

gutSMASH version 1.0 presents a first step to mine anaerobic genomes for primary metabolic gene clusters and bioenergetics, but we already anticipate that, in the near future, a more updated version of the tool will have to include new detection rules to predict newly characterized gene clusters. For this reason, users are advised to regularly check the online gutSMASH documentation for a description of the most up-to-date list of detected gene cluster types. In the future, a comprehensive database of gutSMASH-predicted gene clusters, similar to the antiSMASH database for antiSMASH-detected gene clusters (27), would also be useful to allow users to query the database based on taxonomic entities of interest (e.g., at the species or genus level) or retrieve gene clusters with specific combinations of protein families of interest. Overall, we believe that with this work we provide the community with a useful asset to profile microbiomes for specialized primary MGCs and bioenergetics to better understand the chemistry in this ecosystem with the benefit of a ready-to-use streamlined protocol.

## DATA AVAILABILITY

The source code for the gutSMASH software hosted by the web server can be found at https://github.com/victoriapascal/gutsmash.
